# Storage duration of vitrified embryos does not affect pregnancy and neonatal outcomes after frozen-thawed embryo transfer

**DOI:** 10.3389/fendo.2023.1148411

**Published:** 2023-03-14

**Authors:** Xuelan Li, Pingping Guo, Christophe Blockeel, Xinning Li, Ling Deng, Jie Yang, Chujun Li, Min Lin, Haocun Wu, Guifeng Cai, Yunzhao Hu, Xin Chen

**Affiliations:** ^1^ Reproductive Medicine Center, Shunde Hospital of Southern Medical University (The First People’s Hospital of Shunde), Foshan, Guangdong, China; ^2^ Reproductive Medicine Center, Zhuhai Maternal and Childcare Service Center, Zhuhai, Guangdong, China; ^3^ Centre for Reproductive Medicine, Universitair Ziekenhuis Brussel, Vrije Universiteit Brussel, Brussels, Belgium; ^4^ Department of Clinical Laboratory, Shunde Hospital of Southern Medical University (The First People’s Hospital of Shunde), Foshan, Guangdong, China

**Keywords:** cryopreservation, vitrification, frozen embryo transfer, storage time, pregnancy outcomes

## Abstract

**Background:**

With the refinement of cryopreservation technology, the number of frozen-warmed embryo transfer (FET) cycles and cryopreserved embryos has increased rapidly. However, studies investigating the effect of storage duration on pregnancy outcomes after vitrification are limited and their results are controversial. Furthermore, the available studies did not take patients’ demographic nor clinical treatment characteristics into account and the cryo-storage duration was short. So this study aimed to explore the effect of storage duration of vitrified warmed embryos on pregnancy and neonatal outcomes in patients with good prognosis and long storage duration of vitrified embryos.

**Methods:**

This study was a bi-centre, retrospective study including 1037 women undergoing their first FET cycles following a fresh cycle from January 2012 until December 2021. Patients were divided into four storage groups in accordance with the storage duration of transferred embryos (612 patients in group I, with storage duration between 1 and 6 months; 202 patients in group II, with storage duration between 7 and 12 months; 141 patients in group III, with storage duration between 13 and 36 months; and 76 patients in group IV, with storage duration between 37 and 84 months). The pregnancy and neonatal outcomes were compared amongst different storage duration groups.

**Results:**

Amongst the different groups, no significant differences were observed in the pregnancy outcomes, including biochemical pregnancy rate, implantation rate, clinical pregnancy rate, ongoing pregnancy rate and live birth rate. In addition, no evidence of differences amongst different storage duration groups was observed in terms of preterm birth, birth length and low birthweight.

**Conclusions:**

The pregnancy and neonatal outcomes of embryos after vitrification were not impaired by storage duration up to 7 years.

## Introduction

Since the first clinical pregnancy from frozen-warmed embryo transfer (FET) was reported in 1983 (1), cryopreservation techniques have been routinely performed nowadays to store gametes and embryos. With the refinement of cryopreservation technology, especially embryo vitrification, pregnancy rates after FET are now equal to or even better than pregnancy rates after fresh transfer (2, 3). FET cycles do not only improve the cumulative pregnancy rate from a single oocyte retrieval cycle (4) but also significantly reduce the risk of moderate to severe ovarian hyperstimulation syndrome (5). Moreover, current evidence suggests that compared with fresh embryos, pregnancies conceived from frozen embryos are associated with decreased risks of small for gestational age, low birthweight and preterm delivery (6, 7). Consequently, the number of FET cycles has increased rapidly. In China, the number of FET cycles increased from 81,929 in 2013 to 151,889 in 2016 (8). In Europe, the proportion of cryopreserved embryo transfers is even higher than 60% in countries such as Switzerland, Finland, the Netherlands and Iceland (9).

With the increase in cryopreserved embryos, some researchers have investigated the possible damage or injury to embryos after cryopreservation, such as toxic or non-specific effects of cryoprotectants and free radicals (10). However, studies on the simulated cumulative effect of background radiation during storage failed to determine any detrimental effect when frozen mouse embryos were exposed to the equivalent of about 2000 years of background radiation (11). Some animal studies have indicated that with the storage duration of mouse embryos increased, the post-thaw survival rate decreased and the chromosomal aberration increased accordingly (12, 13). However, other animal studies demonstrated that embryonic and pregnancy outcomes were not significantly affected by the storage duration of embryos (14–17). Consistent with the animal studies, the human population studies evaluating the effect of cryopreservation storage duration on FET outcomes are still a matter of debate. Some studies found that cryopreservation did not affect clinical, obstetric and perinatal outcomes (18–24). However, other large retrospective cohort studies suggested that the prolonged cryo-storage duration of embryos negatively affected pregnancy outcomes (25–28). These contradictory results indicate that whether prolonged cryo-storage affects the embryonic or clinical outcomes remains unknown.

As the Chinese government announced the two-child policy in 2015, according to a national cross-sectional survey, the fertility intention to have a second child was about 60% amongst the Chinese population (29). Meanwhile, with the increase in cryopreserved embryos and the trend of delayed childbearing in many industry countries, an increasing number of cryopreserved embryos are transferred in the future so drawing conclusions about the effect of long cryo-storage duration on human embryos is of great importance. Thus, this retrospective cohort study was conducted to investigate the effect of storage duration of vitrified warmed embryos on reproductive outcomes amongst 1037 women who underwent their first FET cycle following fresh embryo transfer.

## Materials and methods

### Study design and participants

This bi-centre retrospective study was carried out in the Reproductive Medicine Center, Shunde Hospital of Southern Medical University (The First People’s Hospital of Shunde) & Reproductive Medicine Center, Zhuhai Maternal and Childcare Service Center. In total, 1037 women who underwent their first FET cycle following a fresh cycle transfer from January 2012 to December 2021 were included. Patients who were under 40 years old at oocyte retrieval (OR) and had at least one good-quality embryo transferred at the FET cycle were included in the study. The exclusion criteria were as follows: i. no viable embryos available for transfer after vitrification; ii. more than two previous ORs; iii. recurrent implantation failure [RIF, defined as failure to achieve clinical pregnancy after transfer of at least four good-quality embryos in a minimum of three fresh or frozen cycles in a woman under the age of 40 years (30)]; iv. decreased ovarian reserve, defined as antral follicle count (AFC) < 5–7 or anti-Mullerian hormone (AMH) < 1.1 ng/mL; v. uterine anomalies (unicornuate uterus, didelphys uterus and septate uterus); vi. severe intrauterine adhesions, according to the Chinese experts’ consensus on clinical diagnosis and treatment of intrauterine adhesions (31); vii. recurrent spontaneous abortion or with adverse pregnancy histories; viii. chromosomal abnormality. Approval for this study was obtained from the Ethical Committee of Shunde Hospital, Southern Medical University (20210301).

Patients were divided into four storage groups in accordance with the storage duration of the transferred embryos: group I, with a storage duration of 1–6 months; group II, 7–12 months; group III: 13–36 months; and group IV, 37–84 months.

### Procedures

The ART procedures, including ovarian stimulation, oocyte retrieval, insemination, embryo freezing and embryo transfer occurred in accordance with the standard procedure. The patients were given either a gonadotrophin releasing hormone (GnRH) antagonist protocol or a long GnRH agonist protocol for ovarian stimulation. Oocyte retrieval was performed 34–36 h after triggering with hCG, GnRH agonist or combined hCG and GnRH agonist by experienced physicians. Oocytes were fertilised by either conventional *in-vitro* fertilisation (IVF) or intracytoplasmic sperm injection (ICSI). Normal fertilisation was confirmed by the presence of two pronuclei at 16–18 h after conventional IVF or ICSI. Embryos with 7–9 cells, equal size, regular shape and < 20% fragmentation were considered as good-quality embryos. Those with at least five cells and < 20% fragmentation were vitrified. Blastocyst quality was assessed in accordance with the degree of blastocoele expansion and the quality of the inner cell mass and trophectoderm, which were classified in accordance with the Gardner blastocyst grading scale (32). High-quality blastocyst was defined as that of grade ≥ 3BB.

Cleavage-stage embryos were vitrified on day 3 (D3) and blastocysts were vitrified on day 5 or 6 on the basis of embryonic development. The vitrification/thawing procedure was performed using a vitrification/thawing kit from JieYing Laboratory Inc. (Canada) before December 2013, and a vitrification/thawing kit from Kitazato Corporation (Japan) since January 2014. The operation procedures were performed in accordance with the manufacturer’s instructions. For the vitrification procedure, the embryos were transferred to equilibration solution (ES) for 5min (JieYing kit)/5–15 min (Kitazato kit) at room temperature and then to vitrification solution (VS), equilibrated for 1 min (JieYing kit)/30–60 s (Kitazato kit), loaded into straws with minimal volume of VS and then plunged immediately into liquid nitrogen for storage. For the thawing process of vitrified embryos, thawing solutions (TSs) 1–4 were firstly warmed to room temperature (JieYing Kit), TS was warmed to 37°C and diluent solution (DS) and washing solution (WS) were warmed to room temperature (Kitazato Kit). Secondly, the embryos unloaded from the carriers were immediately submerged into TS1 (JieYing kit)/TS (Kitazato kit) and gently washed for 1 min. Thirdly, the warmed embryos were transferred to TS2 (JieYing kit)/DS (Kitazato kit) for 3 min, TS3(JieYing kit)/WS (Kitazato kit) for 5 min and then TS4 (JieYing kit)/another WS (Kitazato kit) for 5 min. After washing, the embryos were transferred to an incubator for culture to complete recovery. The embryologists have undergone strict training to perform vitrification technically. The have also undergone annual quality control assessment. The liquid nitrogen was refilled regularly every week. The laboratory procedures remained unchanged throughout the study period in the two centres. The same storage tanks and pieces of technical equipment were used over the years included in the study period.

No assisted hatching on D3 embryo or blastocyst was performed. The embryos were thawed at an appropriate time in accordance with the individual transfer protocol and then cultured until transfer. FET was performed following endometrial preparation by natural monitoring, an ovarian stimulation cycle or hormone replacement therapy. Serum β-hCG test was performed on day 14 after embryo transfer.

In the last few years, the transfer of fewer embryos was emphasised throughout the world. Since 2017, the clinical practices in both centres directing the number of transferred embryos have recommended a maximum of two embryos to any patient regardless of prognosis or circumstance. Further, culturing all cleavage embryos to the blastocyst stage has gained popularity since 2017 in both centres. These changes in policy were critical in successfully lowering the incidence of multiple pregnancies in good-prognosis patients whilst still establishing pregnancies in older patients.

### Outcomes

The primary outcome was live birth rate and the secondary outcomes included biochemical pregnancy rate, clinical pregnancy rate, ongoing pregnancy rate, multiple pregnancy rate, ectopic pregnancy rate, miscarriage rate, gestational age, birth length and weight of newborns and birth defects. Biochemical pregnancy was defined as serum β-hCG level > 5 mIU/ml. Clinical pregnancy was defined as the presence of an intrauterine gestational sac by transvaginal ultrasound at 6 gestational weeks and ectopic pregnancy was considered as an ultrasound documented gestational sac outside the uterine cavity or pathologic evidence of an extrauterine pregnancy. Ongoing pregnancy rate was defined as detection of a viable foetus with foetal heartbeat beyond 12 weeks of gestation. Miscarriage was defined as clinical pregnancy losses prior to 28 weeks of gestation. Live birth was defined as the delivery of a live-born infant. Only one live birth was counted when twins or higher-order multiple births were delivered. The association of storage duration with neonatal outcomes was evaluated in singletons. The neonatal outcomes were gestational age, birthweight, preterm birth (gestation < 37 weeks), low birthweight (birthweight < 2500 g) and macrosomia (birthweight > 4000 g). Birth defects were defined in accordance with the International Classification of Diseases, 10th Revision (ICD-10), following Zhu’s study (33).

### Statistical analysis

The baseline characteristics and clinical outcomes were described as mean ± SD for continuous variables and as frequency with proportion for categorical variables. For continuous variables, differences between groups were compared using ANOVA when the homogeneity of variances assumption was met; otherwise, Welch’s test was used. For categorical variables, Pearson’s chi-square test or Fisher’s exact test was used as appropriate. All statistical analyses were performed using SPSS version 21.0 (SPSS Inc., Chicago, IL), with *P* < 0.05 considered statistically significant.

## Results

Between January 2012 and December 2021, 1037 women were included in the study (893 women were recruited from the Zhuhai centre and 144 women from the Shunde centre). Amongst them, 1031 patients who underwent their first frozen embryo transfer following fresh embryo transfer met the inclusion and exclusion criteria and they were grouped in accordance with storage duration. In groups I–IV, 612, 202, 141 and 76 patients were included, respectively. Six patients with storage duration between 85 and 115 months were not included in the above groups owing to small sample size.

The baseline and fresh cycle treatment characteristics of different storage duration groups are summarised in **Table 1**. Significant differences were found across the groups in the characteristics age at OR, baseline AFC and total Gn dose. However, difference was observed in the estradiol level on the trigger day, number of retrieved oocytes, number of available or good quality embryos on D3. The number of transferred and good-quality transferred embryos in the previous cycles were statistically significantly different amongst the groups. No significant differences were observed amongst the four groups in terms of other baseline or treatment characteristics, including body mass index, duration of infertility, primary infertility proportion, baseline FSH, fertilisation method and normal fertilisation rate (**Table 1**).

**Table 1:** Baseline and fresh cycle treatment characteristics of the study population in different storage duration groups.

Storage Groups	I	II	III	IV	*P*-value
Storage duration (months)	1-6	7-12	13-36	37-84	
No. of cycles	612	202	141	76	
Age at OPU (years)	31.86 ± 3.72	31.93 ± 3.55	31.30 ± 3.58	29.49 ± 2.82	P<0.001
Body mass index (kg/m²)	21.76 ± 3.36	21.88 ± 3.41	21.74 ± 2.95	21.18 ± 2.45	0.446
Primary infertility	270 (44.1%)	97 (48.0%)	73 (51.8%)	35 (46.1%)	0.377
Duration of infertility (years)	3.79 ± 2.62	4.13 ± 3.15	3.87 ± 2.79	3.57 ± 2.84	0.364
bFSH (IU/L)	6.72 ± 2.24	6.54 ± 1.94	6.54 ± 1.77	6.68 ± 1.72	0.657
AFC	14.66 ± 5.30	15.34 ± 5.21	16.23 ± 5.10	17.80 ± 5.35	P<0.001
Previous embryos transferred	2.09 ± 0.90	2.23 ± 0.91	2.28 ± 0.86	2.33 ± 1.05	0.020
Previous good-quality embryos transferred	1.90 ± 0.71	2.04 ± 0.66	2.03 ± 0.65	2.16 ± 0.57	0.001
Total gonadotropin dose (IU)	2580.45± 899.10	2539.25± 983.77	2356.38± 915.70	2201.65± 873.80	0.001
Estradiol level on HCG trigger day (pmol/L)	12936.93± 10029.92	11841.97± 8047.12	11873.93± 9155.30	13662.59± 11418.36	0.276
Number of oocytes retrieved	11.39 ± 4.09	11.40 ± 4.01	11.01 ± 3.66	11.38 ± 4.11	0.786
Fertilization method					
IVF	452 (73.9%)	143 (70.8%)	102 (72.3%)	56 (73.7%)	0.855
ICSI	120 (19.6%)	47 (23.3%)	25 (17.7%)	17 (22.4%)	0.560
IVF+ICSI	2 (0.3%)	2 (1.0%)	0 (0.0%)	0 (0.0%)	0.484
Normal fertilization rate (%)	72.80 ± 16.53	72.71 ± 17.66	73.87 ± 15.64	73.99 ± 16.02	0.848
Number of available embryos on D3	6.39 ± 2.47	6.32 ± 2.20	6.40 ± 2.28	6.26 ± 2.13	0.958
Number of good quality embryos on D3	4.10 ± 1.85	4.02 ± 1.68	4.29 ± 1.80	4.24 ± 1.67	0.515

bFSH, baseline follicle stimulating hormone. Data are mean ± SD or n (%).

In the FET treatment cycle, with longer storage duration, the maternal age at FET increased (*P* < 0.05) and the proportion of transferred cleavage embryos gradually increased (*P* < 0.05). Correspondingly, the proportion of transferred blastocysts decreased (*P* < 0.05). No significant differences were observed in the number of transferred and good-quality transferred embryos. Moreover, the live birth rates in groups I–IV were 40.7%, 41.1%, 39.7% and 39.5% (*P >* 0.05), respectively. No significant differences were observed amongst the groups regarding other pregnancy outcomes, including biochemical pregnancy rate, implantation rate, clinical pregnancy rate, ongoing pregnancy rate, miscarriage rate, multiple pregnancy rate and ectopic pregnancy rate (**Table 2**). The clinical outcomes of FETs with storage duration > 3 years are shown in **Table 3**. Only six FET cycles had storage duration > 7 years, of which none resulted in pregnancy.

**Table 2:** Clinical outcomes following transfer of vitrified embryos in different storage duration groups.

Storage Groups	I	II	III	IV	*P*-value
Storage duration (months)	1-6	7-12	13-36	37-84	
Mean storage duration (months)	3.59±1.48	8.93±1.74	23.24±7.42	50.83±11.73	
Age at FET (years)	31.90±3.71	32.89±3.61	33.20±3.65	33.68±3.10	P<0.001
Endometrial thickness (mm)	9.81±1.72	9.54±1.90	9.85±1.82	9.82±1.88	0.282
Number of embryos transferred	1.74±0.75	1.79±0.77	1.80±0.72	1.71±0.65	0.613
Number of good-quality embryos transferred	1.41±0.59	1.45±0.62	1.46±0.62	1.37±0.51	0.555
Development stage of embryos transferred					
Cleavage embryo	289 (47.2%)	108 (53.5%)	80 (56.7%)	54 (71.1%)	P<0.001
Blastocyst	323 (52.8%)	94 (46.5%)	61 (43.3%)	22 (28.9%)	P<0.001
Biochemical pregnancy rate	49 (8.0%)	14 (6.9%)	7 (5.0%)	3 (3.9%)	0.408
Implantation rate	366/1062 (34.5%)	124/362 (34.3%)	81/254 (31.9%)	51/130 (39.2%)	0.560
Clinical pregnancy rate	308 (50.3%)	104 (51.5%)	68 (48.2%)	39 (51.3%)	0.943
Ongoing pregnancy rate	258 (42.2%)	89 (44.1%)	59 (41.8%)	32 (42.1%)	0.967
Live birth rate	249 (40.7%)	83 (41.1%)	56 (39.7%)	30 (39.5%)	0.991
Miscarriage rate	48 (15.6%)	17 (16.3%)	10 (14.7%)	5 (12.8%)	0.959
Multiple pregnancy rate	62 (20.1%)	20 (19.2%)	13 (19.1%)	12 (30.8%)	0.437
Ectopic pregnancy rate	8 (2.6%)	2 (1.9%)	0 (0.0%)	2 (5.1%)	0.322

Data are mean±SD, n (%) or n/N (%).

**Table 3:** Clinical outcomes of FETs with storage duration greater than 3 years.

Storage duration (years)	Storage duration (months)	No. of FET cycles	No. of embryos transferred	No. of good quality embryos transferred	Implantation rate	Clinical pregnancy rate	Live birth rate
3-4	37-48	43	1.77 ± 0.68	1.40 ± 0.54	33/76 (43.4%)	23 (53.5%)	17 (39.5%)
4-5	49-60	18	1.50 ± 0.62	1.22 ± 0.43	7/27 (25.9%)	6 (33.3%)	4 (22.2%)
5-7	61-84	15	1.80 ± 0.56	1.47 ± 0.52	11/27 (40.7%)	10 (66.7%)	9 (60.0%)
7-10	85-120	6	2.50 ± 0.84	1.83 ± 0.75	0/15 (0.0%)	0 (0.0%)	0 (0.0%)

Data are mean ± SD or n/N (%).

Subgroup analysis was conducted to evaluate the effect of storage duration of vitrified warmed embryos on pregnancy outcomes at the cleavage and blastocyst stages to avoid bias caused by the transferred embryos in different development stages. At the cleavage stage, the maternal age at FET increased with longer storage duration (*P* < 0.05). The endometrial thickness was not statistically different amongst the groups. The number of cleavage transferred and good-quality cleavage transferred embryos in group IV were significantly lower than in the other groups. However, no significant differences were observed amongst the groups in terms of pregnancy outcomes, including biochemical pregnancy rate, implantation rate, clinical pregnancy rate, ongoing pregnancy rate, live birth rate, miscarriage rate, multiple pregnancy rate and ectopic pregnancy rate (**Table 4**). At the blastocyst stage, the baseline characteristics and pregnancy outcomes of the FET cycle amongst the four groups did not show any significant differences, except the maternal age at FET, which still increased with longer storage duration (*P* < 0.05, **Table 5**).

**Table 4:** Clinical outcomes following transfer of vitrified cleavage embryos in different storage duration groups.

Storage Groups	I	II	III	IV	*P*-value
Storage duration (months)	1-6	7-12	13-36	37-84	
No. of cycles	289	108	80	54	
Mean storage duration (months)	3.63 ± 1.50	9.06 ± 1.75	23.36 ± 7.62	52.22 ± 11.86	
Age at FET (years)	31.93 ± 3.75	32.42 ± 3.43	33.15 ± 3.65	33.69 ± 3.00	0.002
Endometrial thickness (mm)	9.92 ± 1.81	9.43 ± 1.83	9.96 ± 1.88	9.98 ± 1.86	0.081
Number of embryos transferred	2.31 ± 0.62	2.33 ± 0.60	2.25 ± 0.56	1.91 ± 0.62	P<0.001
Number of good-quality embryos transferred	1.73 ± 0.65	1.79 ± 0.64	1.73 ± 0.66	1.48 ± 0.54	0.032
Biochemical pregnancy rate	14 (4.8%)	3 (2.8%)	1 (1.3%)	3 (5.6%)	0.412
Implantation rate	155/667 (23.2%)	63/252 (25.0%)	43/180 (23.9%)	32/103 (31.1%)	0.384
Clinical pregnancy rate	118 (40.8%)	51 (47.2%)	34 (42.5%)	22 (40.7%)	0.710
Ongoing pregnancy rate	93 (32.2%)	44 (40.7%)	27 (33.8%)	19 (35.2%)	0.461
Live birth rate	91 (31.5%)	42 (38.9%)	26 (32.5%)	18 (33.3%)	0.579
Miscarriage rate	20 (16.9%)	6 (11.8%)	8 (23.5%)	2 (9.1%)	0.395
Multiple pregnancy rate	37 (31.4%)	12 (23.5%)	9 (26.5%)	9 (40.9%)	0.462
Ectopic pregnancy rate	7 (5.9%)	2 (3.9%)	0 (0.0%)	1 (4.5)	0.600

Data are mean ± SD, n (%) or n/N (%).

**Table 5:** Clinical outcomes following transfer of vitrified blastocysts in different storage duration groups.

Storage groups	I	II	III	IV	*P*-value
Storage duration (months)	1-6	7-12	13-36	37-84	
No. of cycles	323	94	61	22	
Mean storage duration (months)	3.56 ± 1.46	8.79 ± 1.74	23.08 ± 7.20	47.41 ± 10.91	
Age at FET (years)	31.87 ± 3.69	33.43 ± 3.75	33.28 ± 3.67	33.68 ± 3.41	P<0.001
Endometrial thickness (mm)	9.71 ± 1.63	9.68 ± 1.98	9.70 ± 1.74	9.41 ± 1.92	0.894
Number of blastocyst transferred	1.22 ± 0.42	1.17 ± 0.38	1.21 ± 0.41	1.23 ± 0.43	0.744
Number of good-quality blastocyst transferred	1.11 ± 0.32	1.06 ± 0.25	1.11 ± 0.32	1.09 ± 0.29	0.427
Biochemical pregnancy rate	35 (10.8%)	11 (11.7%)	6 (9.8%)	0 (0.0%)	0.422
Implantation rate	211/395 (53.4%)	61/110 (55.5%)	38/74 (51.4%)	19/27 (70.4%)	0.354
Clinical pregnancy rate	190 (58.8%)	53 (56.4%)	34 (55.7%)	17 (77.3%)	0.313
Ongoing pregnancy rate	165 (51.1%)	45 (47.9%)	32 (52.5%)	13 (59.1%)	0.802
Live birth rate	158 (48.9%)	41 (43.6%)	30 (49.2%)	12 (54.5%)	0.745
Miscarriage rate	28 (17.7.%)	11 (20.8%)	2 (5.9%)	3 (17.6%)	0.295
Multiple pregnancy rate	25 (13.2%)	8 (15.1%)	4 (11.8%)	3 (17.6%)	0.901
Ectopic pregnancy rate	1 (0.5%)	0 (0.0%)	0 (0.0%)	1 (5.9%)	0.199

Data are mean ± SD, n (%) or n/N (%).

Amongst all singletons born after FET, the neonatal outcomes of groups–IV were analysed (**Table 6**). None of the groups showed birth defects diagnosed in singletons and multiples. The proportion of macrosomia in group IV was higher than that in other groups, without significant difference. Moreover, no evidence of differences was found across all groups in terms of preterm birth, birth length and low birthweight.

**Table 6:** Neonatal outcomes of singletons born after frozen embryo transfer in different storage duration groups.

Storage groups	I	II	III	IV	*P*-value
Storage duration (months)	1-6	7-12	13-36	37-84	
Live birth babies	293	94	65	40	
Singletons	205	72	47	20	
Multiples	88	22	18	20	
Preterm Proportion	14 (6.8%)	6 (8.3%)	1 (2.1%)	1 (5.0%)	0.601
Birth length (cm)	49.93 ± 2.02	49.86 ± 1.44	50.83 ± 2.04	49.93 ± 4.11	0.185
Low birth weight	10 (4.9%)	4 (5.6%)	1 (2.1%)	1 (5.0%)	0.829
Macrosomia	8 (3.9%)	4 (5.6%)	3 (6.4%)	3 (15.0%)	0.156

Data are mean ± SD, n (%) or n/N (%).

## Discussion

To the best of the authors’ knowledge, this study was the first to explore the effect of storage duration of vitrified embryos on pregnancy and neonatal outcomes in patients with good prognosis and long storage duration of vitrified embryos. In this study, the pregnancy and neonatal outcomes of embryos after vitrification were not impaired by storage duration up to 7 years.

Although the landscape of cryopreservation changed dramatically in recent years, the studies evaluating the effect of storage duration of vitrified warmed embryos on FET outcomes were still limited. The first study to evaluate the effect of extended storage duration of vitrified blastocysts on embryonic and clinical outcomes was that of Wirleitner’s, who included the transfer of blastocysts that were vitrified aseptically using a closed system (24). Ueno’s and Li’s studies focused on the long-term storage of embryos vitrified using an open device vitrification system (23, 34). They found that long-term storage of vitrified embryos had no negative effect on pregnancy outcomes, consistent with the results of the study. However, the above studies did not consider patients’ demographic or clinical treatment characteristics. Meanwhile, some large retrospective studies conducted in China in different centres drew the similar conclusion that prolonged cryopreservation negatively affected pregnancy outcomes (25–28), thus raising a warning for the scientific community. However, the baseline and clinical information of these studies showed a high heterogeneity amongst different storage groups, which could introduce unavoidable bias although relevant confounding adjustment or stratification analysis was performed. Considering the conclusion of Li’s study (26), Parmegiani and Vajta also raised the same perplexity of whether the progressive decrease in pregnancy and implantation rates is related to cryo-storage duration or patient’s age, basal characteristics and cryo-storage conditions (35). Furthermore, all patients included in these studies underwent their first FET cycles following an freeze-all strategy, thereby explaining why the maximum storage duration in these studies was short as patients usually returned to FET treatment in a short time after the freeze-all strategy. Thus, the results of these studies could only reflect the possible effect of short- to medium-term embryo cryopreservation on FET outcomes.

In contrast to the above studies, the present study set strict inclusion and exclusion criteria so the included patients had mostly good prognosis. As a consequence, slight difference was observed in the demographic or clinical characteristics. Although significant differences were found amongst the groups in terms of age at OR, AFC and total Gn dose, the Gn dose increased when AFC decreased so no difference was seen in the number of retrieved oocytes and available or good-quality embryos on D3 in the fresh cycle. Secondly, patients who underwent their first FET cycle after the fresh embryo transfer were included because some patients returned to FET treatment in the hope of having a second FET-conceived child. Thus, the cryo-storage duration of vitrified embryos in this study was much longer than that in other studies, making the results more convincing.

With longer storage duration, the proportion of cleavage transferred embryo gradually increased and that of transferred blastocyst decreased. This observation could be explained by the fact that culturing all cleavage embryos to the blastocyst stage has gained popularity within the past 5 years in both centres. So a subgroup analysis was conducted to adjust for the potential confounding factor of different development stages of transferred embryos. The results showed no adverse effect of storage duration of vitrified embryos on pregnancy outcomes at the cleavage and blastocyst stages.

In this study, only six FET cycles had storage duration > 7 years and no pregnancies were observed. Therefore, the safety of long-term cryo-storage of human embryos beyond 7 years could not be ensured given the limited data. To date, the maximum length of embryo storage duration remains unclear. According to Mazur et. al, when the temperature is below -130°C, many cells could stay stable for ages because the enzyme activity in the cell in liquid nitrogen is almost completely suppressed (36). In a retrospective observational study, a group of 20 patients and a total of 28 FET cycles with embryo storage duration between 12 and 17.1 years were followed. The clinical pregnancy rate and the live birth rate of these long-term cryopreserved embryos after slow freezing were 30.43% and 21.74%, respectively. However, the study did not mention the quality of the transferred embryo so whether the low live birth rate of these patients was due to the long-term cryopreservation or other reasons remained unknown (37). The record for the longest embryo stored before transfer and giving birth is 27 years (38). Other studies about the effect of human embryos cryopreserved for over 10 years were almost case reports (20, 39, 40). Therefore, a solid clinical evidence to draw conclusions on the safety of long-term cryo-storage of human embryos is lacking.

Previous studies have reported that embryo cryopreservation resulted in an increased risk of high birthweight and showed that the potential cause of this increase was related to epigenetic modifications (41–43). The only study that examined DNA integrity in human embryos suggested that vitrification affects DNA integrity to a much lesser extent than slow freezing (44). Furthermore, through single-cell RNA-Seq, a total of 128 mRNAs and 365 lncRNAs in vitrified-warmed embryos were found to be differentially expressed compared with those in fresh embryos. However, the transcriptome between embryos cryostored for 3 and 8 years was not differentially expressed, which indicated that long-term storage after vitrification does not affect the efficacy and safety of human embryos. Nevertheless, the procedure of vitrification warming could lead to minor alteration in transcriptome (45). So the proportion of low birthweight, high birthweight, macrosomia and other adverse neonatal outcomes did not change significantly with the length of storage duration in previous studies (23, 26), consistent with the results of the present study.

This study also have several limitations. Firstly, the sample size was small, especially in the storage group with a longer embryo cryo-storage duration. Therefore, solid conclusions were not drawn. Secondly, the retrospective study design may have increased the risk of bias in patient selection although strict inclusion and exclusion criteria were set. More studies are required to further investigate the effect of long-term embryo cryo-storage on pregnancy and neonatal outcomes.

## Conclusion

In summary, the results of this strictly controlled retrospective cohort study suggested that the pregnancy and neonatal outcomes of embryos after vitrification were not impaired by storage duration up to 7 years. With the rapid development of cryopreservation technology and the increasing demand for fertility preservation in recent years, more studies could be required to further investigate the effect of long term embryos cryopreservation on embryonic or clinical outcomes.

## Data availability statement

The original contributions presented in the study are included in the article/supplementary material. Further inquiries can be directed to the corresponding authors.

## Ethics statement

The approval for this study was obtained from the ethical committee of Shunde Hospital, Southern Medical University (20210301). The patients/participants provided their written informed consent to participate in this study.

## Author contributions

XLL and XC: design of the study, analysis and interpretation of data, and revise the article. CB: design of the study and revise the article. PG, YH and GC: acquisition of data, drafting the article. ML, JY, CL, LD: revising the article, analysis and interpretation of data. PG, XNL and HW: collection of data. All authors approved the final version of the manuscript.

## References

[1] Trounson A Mohr L. Human pregnancy following cryopreservation, thawing and transfer of an eight-cell embryo. Nature (1983) 305:707–9. doi: 10.1038/305707a0 6633637

[2] Bosdou JK Venetis CA Tarlatzis BC Grimbizis GF Kolibianakis EM. Higher probability of live-birth in high, but not normal, responders after first frozen-embryo transfer in a freeze-only cycle strategy compared to fresh-embryo transfer: a meta-analysis. Hum Reprod (2019) 34:491–505. doi: 10.1093/humrep/dey388 30689865

[3] Wei D Liu JY Sun Y Shi Y Zhang B Liu JQ. Frozen versus fresh single blastocyst transfer in ovulatory women: a multicentre, randomised controlled trial. Lancet (2019) 393:1310–8. doi: 10.1016/S0140-6736(18)32843-5 30827784

[4] De Geyter C Calhaz-Jorge C Kupka MS Wyns C Mocanu E Motrenko T. ART in Europe 2013: results generated from European registries by ESHRE. Hum Reprod Open (2020) 2020):hoz038. doi: 10.1093/hropen/hoz038 32995563 PMC7508022

[5] Zhu Q Chen Q Wang L Lu X Lyu Q Wang Y. Live birth rates in the first complete IVF cycle among 20 687 women using a freeze-all strategy. Hum Reprod (2018) 33:924–9. doi: 10.1093/humrep/dey044 29534178

[6] Conforti A Picarelli S Carbone L La Marca A Venturella R Vaiarelli A. Perinatal and obstetric outcomes in singleton pregnancies following fresh versus cryopreserved blastocyst transfer: a meta-analysis. Reprod BioMed Online (2021) 42:401–12. doi: 10.1016/j.rbmo.2020.09.029 33234401

[7] Maheshwari A Pandey S Amalraj RE Shetty A Hamilton M Bhattacharya S. Is frozen embryo transfer better for mothers and babies? can cumulative meta-analysis provide a definitive answer? Hum Reprod Update (2018) 24:35–58. doi: 10.1093/humupd/dmx031 29155965

[8] Hu L Bu Z Huang G Sun H Deng C Sun Y. Assisted reproductive technology in China: Results generated from data reporting system by CSRM from 2013 to 2016. Front Endocrinol (Lausanne) (2020) 11:458. doi: 10.3389/fendo.2020.00458 33042000 PMC7527788

[9] Calhaz-Jorge C De Geyter C Kupka MS de Mouzon J Erb K Mocanu E. Assisted reproductive technology in Europe 2013: results generated from European registers by ESHRE. Hum Reprod (2017) 32:1957–73. doi: 10.1093/humrep/dex264 29117383

[10] Kopeika J Thornhill A Khalaf Y. The effect of cryopreservation on the genome of gametes and embryos: principles of cryobiology and critical appraisal of the evidence. Hum Reprod Update (2015) 21:209–27. doi: 10.1093/humupd/dmu063 25519143

[11] Glenister PH Lyon MF. Long-term storage of eight-cell mouse embryos at - 196 degrees c. J In Vitro Fert Embryo Transf (1986) 3:20–7. doi: 10.1007/BF01131376 3958563

[12] Mozdarani H Moradi SZ. Effect of vitrification on viability and chromosome abnormalities in 8-cell mouse embryos at various storage durations. Biol Res (2007) 40:299–306. doi: 10.4067/S0716-97602007000400004 18449458

[13] Zarei MS Mohseni MA Gourabi H Mozdarani H Mansouri Z. Chromosome abnormalities and viability of vitrified eight-cell mouse embryos at presence of two different cryoprotectants at different storage durations. Cell J (2013) 14:254–63. PMC3593929 23577304

[14] Eum JH Park JK Lee WS Cha KR Yoon TK Lee DR. Long-term liquid nitrogen vapor storage of mouse embryos cryopreserved using vitrification or slow cooling. Fertil Steril (2009) 91:1928–32. doi: 10.1016/j.fertnstert.2008.02.126 18440528

[15] Graves-Herring JE Boone WR. Blastocyst rate and live births from vitrification and slow-cooled two-cell mouse embryos. Fertil Steril (2009) 91:920–4. doi: 10.1016/j.fertnstert.2007.12.045 18314119

[16] Lavara R Baselga M Vicente JS. Does storage time in LN2 influence survival and pregnancy outcome of vitrified rabbit embryos? Theriogenology (2011) 76:652–7. doi: 10.1016/j.theriogenology.2011.03.018 21664667

[17] Sanchez-Osorio J Cuello C Gil MA Parrilla I Almiñana C Caballero I. *In vitro* postwarming viability of vitrified porcine embryos: effect of cryostorage length. Theriogenology (2010) 74:486–90. doi: 10.1016/j.theriogenology.2010.03.003 20452005

[18] Canosa S Cimadomo D Conforti A Maggiulli R Giancani A Tallarita A. The effect of extended cryo-storage following vitrification on embryo competence: a systematic review and meta-analysis. J Assist Reprod Genet (2022) 39:873–82. doi: 10.1007/s10815-022-02405-3 PMC9050987 35119549

[19] Cimadomo D Fabozzi G Dovere L Maggiulli R Albricci L Innocenti F. Clinical, obstetric and perinatal outcomes after vitrified-warmed euploid blastocyst transfer are independent of cryo-storage duration. Reprod BioMed Online (2022) 44:221–7. doi: 10.1016/j.rbmo.2021.09.027 34862135

[20] Dowling-Lacey D Mayer JF Jones E Bocca S Stadtmauer L Oehninger S. Live birth from a frozen-thawed pronuclear stage embryo almost 20 years after its cryopreservation. Fertil Steril (2011) 95:1120–1. doi: 10.1016/j.fertnstert.2010.08.056 20887984

[21] Liu Q Lian Y Huang J Ren X Li M Lin S. The safety of long-term cryopreservation on slow-frozen early cleavage human embryos. J Assist Reprod Genet (2014) 31:471–5. doi: 10.1007/s10815-014-0197-0 PMC3969470 24682782

[22] Riggs R Mayer J Dowling-Lacey D Chi TF Jones E Oehninger S. Does storage time influence postthaw survival and pregnancy outcome? an analysis of 11,768 cryopreserved human embryos. Fertil Steril (2010) 93:109–15. doi: 10.1016/j.fertnstert.2008.09.084 19027110

[23] Ueno S Uchiyama K Kuroda T Yabuuchi A Ezoe K Okimura T. Cryostorage duration does not affect pregnancy and neonatal outcomes: a retrospective single-centre cohort study of vitrified-warmed blastocysts. Reprod BioMed Online (2018) 36:614–9. doi: 10.1016/j.rbmo.2018.03.008 29622403

[24] Wirleitner B Vanderzwalmen P Bach M Baramsai B Neyer A Schwerda D. The time aspect in storing vitrified blastocysts: its impact on survival rate, implantation potential and babies born. Hum Reprod (2013) 28:2950–7. doi: 10.1093/humrep/det361 24030587

[25] Hu KL Hunt S Zhang D Li R Mol BW. The association between embryo storage time and treatment success in women undergoing freeze-all embryo transfer. Fertil Steril (2022) 118:513–21. doi: 10.1016/j.fertnstert.2022.06.003 35934542

[26] Li J Yin M Wang B Lin J Chen Q Wang N. The effect of storage time after vitrification on pregnancy and neonatal outcomes among 24 698 patients following the first embryo transfer cycles. Hum Reprod (2020) 35:1675–84. doi: 10.1093/humrep/deaa136 32575120

[27] Mao Y Tang N Luo Y Yin P Li L. Effects of vitrified cryopreservation duration on IVF and neonatal outcomes. J Ovarian Res (2022) 15:101. doi: 10.1186/s13048-022-01035-8 36076245 PMC9454210

[28] Zhang X Wu S Hao G Wu X Ren H Zhang Y. Prolonged cryopreservation negatively affects embryo transfer outcomes following the elective freeze-all strategy: A multicenter retrospective study. Front Endocrinol (Lausanne) (2021) 12:709648. doi: 10.3389/fendo.2021.709648 34630326 PMC8493094

[29] Jing W Liu J Ma Q Zhang S Li Y Liu M. Fertility intentions to have a second or third child under china's three-child policy: a national cross-sectional study. Hum Reprod (2022) 37:1907–18. doi: 10.1093/humrep/deac101 35554542

[30] Coughlan C Ledger W Wang Q Liu F Demirol A Gurgan T. Recurrent implantation failure: definition and management. Reprod BioMed Online (2014) 28:14–38. doi: 10.1016/j.rbmo.2013.08.011 24269084

[31] Society of Gynaecology and Obstetrics of the Chinese Medical Association. Chinese Experts consensus of clinical diagnosis and treatment of intrauterine adhesions. Chin J Obstet Gynecol (2015) 50:881–7. doi: 10.3760/cma.j.issn.0529-567x.2015.12.001

[32] Gardner DK Lane M Stevens J Schlenker T Schoolcraft WB. Blastocyst score affects implantation and pregnancy outcome: towards a single blastocyst transfer. Fertil Steril (2000) 73:1155–8. doi: 10.1016/s0015-0282(00)00518-5 10856474

[33] Zhu Q Wang N Wang B Wang Y Kuang Y. The risk of birth defects among children born after vitrified blastocyst transfers and those born after fresh and vitrified cleavage-stage embryo transfers. Arch Gynecol Obstet (2018) 298:833–40. doi: 10.1007/s00404-018-4870-x 30135993

[34] Li W Zhao W Xue X Zhang S Zhang X Shi J. Influence of storage time on vitrified human cleavage-stage embryos froze in open system. Gynecol Endocrinol (2017) 33:96–9. doi: 10.1080/09513590.2016.1207166 27449701

[35] Parmegiani L Vajta G. The effect of cryostorage duration on vitrified embryos: has vitrification suddenly become unsafe? Hum Reprod (2020) 35:2632–3. doi: 10.1093/humrep/deaa238 32984877

[36] Mazur P. Freezing of living cells: mechanisms and implications. Am J Physiol (1984) 247:C125–42. doi: 10.1152/ajpcell.1984.247.3.C125 6383068

[37] Yuan Y Mai Q Ma J Deng M Xu Y Zhuang G. What was the fate of human embryos following long-term cryopreservation (≥12 years) and frozen embryo transfer? Hum Reprod (2019) 34:52–5. doi: 10.1093/humrep/dey350 30517678

[38] Honderich H. Baby girl born from record-setting 27-year-old embryo (2022). Available at: https://www.bbc.com/news/world-us-canada-55164607 (Accessed January 4, 2022).

[39] Quintans CJ Donaldson MJ Urquiza MF Carretero I Pasqualini RA Horton M. Live birth of twins after IVF of oocytes that were cryopreserved almost 12 years before. Reprod BioMed Online (2012) 25:600–2. doi: 10.1016/j.rbmo.2012.09.002 23063815

[40] Revel A Safran A Laufer N Lewin A Reubinov BE Simon A. Twin delivery following 12 years of human embryo cryopreservation: case report. Hum Reprod (2004) 19:328–9. doi: 10.1093/humrep/deh046 14747175

[41] Ginström EE Wennerholm UB Khatibi A Petzold M Bergh C. Neonatal and maternal outcome after frozen embryo transfer: Increased risks in programmed cycles. Am J Obstet Gynecol (2019) 221:121–6. doi: 10.1016/j.ajog.2019.03.010 30910545

[42] Litzky JF Boulet SL Esfandiari N Zhang Y Kissin DM Theiler RN. Effect of frozen/thawed embryo transfer on birthweight, macrosomia, and low birthweight rates in US singleton infants. Am J Obstet Gynecol (2018) 218:431–3. doi: 10.1016/j.ajog.2017.12.223 PMC5878119 29291410

[43] Liu SY Teng B Fu J Li X Zheng Y Sun XX. Obstetric and neonatal outcomes after transfer of vitrified early cleavage embryos. Hum Reprod (2013) 28:2093–100. doi: 10.1093/humrep/det104 23569081

[44] Li L Zhang X Zhao L Xia X Wang W. Comparison of DNA apoptosis in mouse and human blastocysts after vitrification and slow freezing. Mol Reprod Dev (2012) 79:229–36. doi: 10.1002/mrd.22018 22213487

[45] Li J Zhu L Huang J Liu W Han W Huang G. Long-term storage does not affect the expression profiles of mRNA and long non-coding RNA in vitrified-warmed human embryos. Front Genet (2021) 12:751467. doi: 10.3389/fgene.2021.751467 35178066 PMC8844023

